# I_Ks_ Protects from Ventricular Arrhythmia during Cardiac Ischemia and Reperfusion in Rabbits by Preserving the Repolarization Reserve

**DOI:** 10.1371/journal.pone.0031545

**Published:** 2012-02-22

**Authors:** Xiaogang Guo, Xiuren Gao, Yesong Wang, Longyun Peng, Yingying Zhu, Shenming Wang

**Affiliations:** 1 Department of Cardiology, The First Affiliated Hospital of Sun Yat-sen University, Guangzhou, China; 2 Intensive Care Unit, Central Hospital, Tai'an, China; 3 Department of Vascular Surgery, The First Affiliated Hospital of Sun Yat-sen University, Guangzhou, China; Tel Aviv University, Israel

## Abstract

**Introduction:**

The function of the repolarization reserve in the prevention of ventricular arrhythmias during cardiac ischemia/reperfusion and the impact of ischemia on slowly activated delayed rectifier potassium current (I_Ks_) channel subunit expression are not well understood.

**Methods and Results:**

The responses of monophasic action potential duration (MAPD) prolongation and triangulation were investigated following an L-768,673-induced blockade of I_Ks_ with or without ischemia/reperfusion in a rabbit model of left circumflex coronary artery occlusion/reperfusion. Ischemia/reperfusion and I_Ks_ blockade were found to significantly induce MAPD90 prolongation and increase triangulation at the epicardial zone at 45 min, 60 min, and 75 min after reperfusion, accompanied with an increase in premature ventricular beats (PVBs) during the same period. Additionally, I_Ks_ channel subunit expression was examined following transient ischemia or permanent infarction and changes in monophasic action potential (MAP) waveforms challenged by β-adrenergic stimulation were evaluated using a rabbit model of transient or chronic cardiac ischemia. The epicardial MAP in the peri-infarct zone of hearts subjected to infarction for 2 days exhibited increased triangulation under adrenergic stimulation. KCNQ1 protein, the α subunit of the I_Ks_ channel, was downregulated in the same group. Both findings were consistent with an increased incidence of PVBs.

**Conclusion:**

Blockade of I_Ks_ caused MAP triangulation, which precipitated ventricular arrhythmias. Chronic ischemia increased the incidence of ventricular arrhythmias under adrenergic stimulation and was associated with increased MAP triangulation of the peri-infarct zone. Downregulation of KCNQ1 protein may be the underlying cause of these changes.

## Introduction

Slowly activated delayed rectifier potassium (I_Ks_) current serves as a repolarizing current in the ventricular cardiomyocytes of humans and various mammals. Together with the rapid (I_Kr_) and inwardly rectifying potassium (I_K1_) current, it provides multiple mechanisms for normal repolarization, which was termed “repolarization reserves” by Roden et al. [1]. Loss of function in one of these currents may not necessarily result in clinical consequences.

Functionally, I_Ks_ constitutes one of the critical repolarization reserves that compensate for reductions in other repolarizing currents, particularly I_Kr_, that are caused by mutations in hereditary long QT syndrome (LQT2) or drugs in acquired LQT syndrome. This is supported by the observation that pharmacological I_Ks_ inhibition played a minor role in the *in vitro* lengthening of action potential duration (APD) in the absence of β-adrenergic stimulation [2], [3]. In contrast, certain drugs, for example sotalol, erythromycin, chlorpromazine, and methadone, or diseases, for example heart failure, diabetes, and cardiac hypertrophy, can trigger a life-threatening arrhythmia in the absence of the repolarization reserve provided by I_Ks_ [4].

Cardiac ischemia and reperfusion are known to change the outward currents responsible for repolarization. For example, when cardiac ischemia was induced, the contribution of adenosine triphosphate sensitive potassium current (I_KATP_) to repolarization was increased, while those of IKr and IK1 were lessened in comparison, which led in turn to a shortening of the APD [5]. The shortening of the APD induced by ischemia is gradually restored by reperfusion, however a temporary prolongation of APD during early reperfusion was observed by Ducroq et al. and Bes et al. in an *in vitro* model of simulated ischemia and reperfusion in isolated cardiomyocytes [6], [7]. This phenomenon, which directly involved repolarization ion current function, has not yet been well explained. In addition, little is currently known about the role played by the repolarization reserve in acute ischemia/reperfusion-induced ventricular arrhythmias. In canine hearts infarcted for 5 days, chronic ischemia decreased I_Ks_ current density and downregulated the expression of KCNQ1 and KCNE1 mRNA [8], [9]. However, the effect of transient and chronic ischemia on the expression of KCNQ1 and KCNE1 proteins has not been determined.

Therefore, the first objective of this study was to evaluate the pattern of I_Ks_ changes during cardiac ischemia and reperfusion, and to determine the function of the repolarization reserve in the prevention of ventricular arrhythmias. In addition, the influence of transient and chronic ischemia on I_Ks_ channel subunit expression and the related electrophysiological outcomes were evaluated.

## Results

### Effect of L-768,673 on ischemia/reperfusion-induced MAP changes

**Figure 1:**
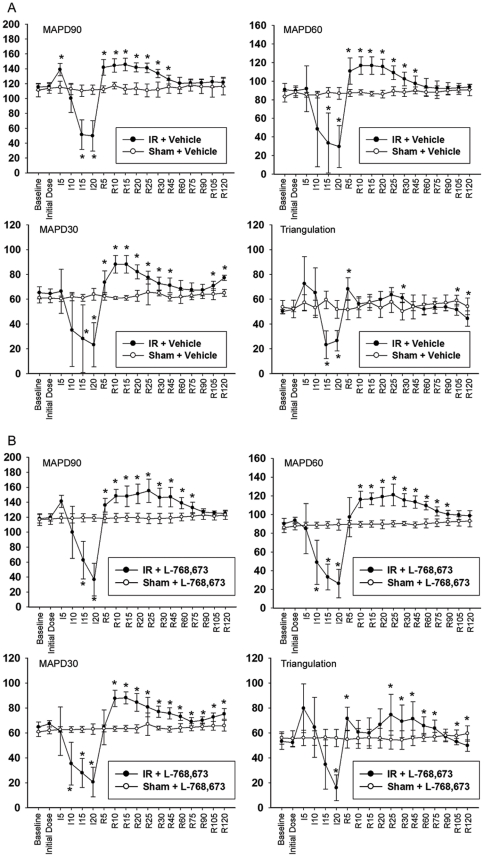
Comparison of epicardial monophasic action potential changes between groups. (**A**) Epicardial monophasic action potential durations (MAPDs) and triangulation (MAPD90–MAPD30) recorded from the ischemia/reperfusion zone (apical) in the Sham+vehicle group and the ischemia/reperfusion (IR)+vehicle group. *P<0.05 vs. Sham+vehicle. (**B**) Epicardial MAPDs and triangulation recorded from the ischemia/reperfusion zone in the Sham+L-768,673 group and the IR+L-768,673 group. *P<0.05 vs. Sham+L-768,673.

In comparison with the Sham+vehicle group, occlusion of the coronary artery greatly shortened the MAP durations at 90%, 60%, and 30% repolarization (MAPD90, MAPD60, and MAPD30) of the ischemic epicardium in the IR+vehicle group; it also caused a decrease in triangulation due to an abrupt shortening of the MAPDs (Figure 1A). When the coronary perfusion was restored, the MAPDs all rebounded and exceeded the baseline value, before they gradually returned to baseline levels with continued reperfusion (Figure 1A). The same phenomena were observed in the IR+L-768,673 group when compared with the Sham+L-768,673 group (Figure 1B).

**Figure 2:**
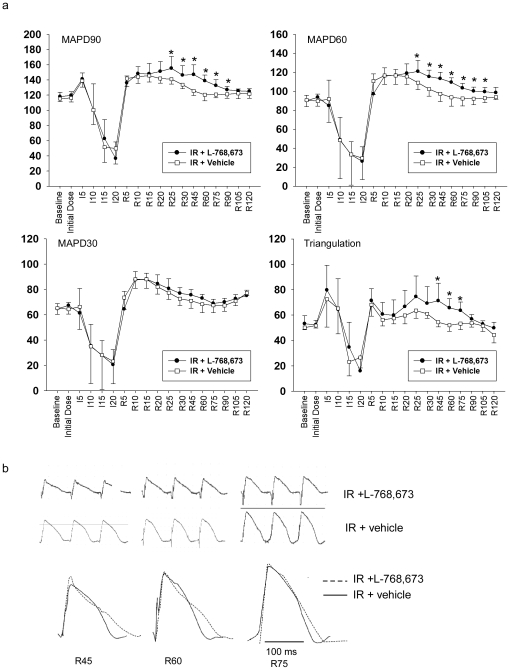
MAPDs, triangulations and MAP waveforms comparison between the IR+L-768,673 and IR+vehicle groups. (**A**) Epicardial MAPDs and triangulation recorded from the ischemia/reperfusion zone (apical) in the IR+L-768,673 group and the IR+vehicle group. Triangulations of the IR+L-768,673 group were increased compared with those of the IR+vehicle group by 31.1%, 26.5%, and 19.3% at R45, R60, and R75 respectively. Results are mean ± standard deviation (STD). * P<0.05 vs. IR+vehicle. (**B**) Comparison of monophasic action potential (MAP) waveforms between the IR+L-768,673 and IR+vehicle groups at R45, R60, and R75.

**Figure 3:**
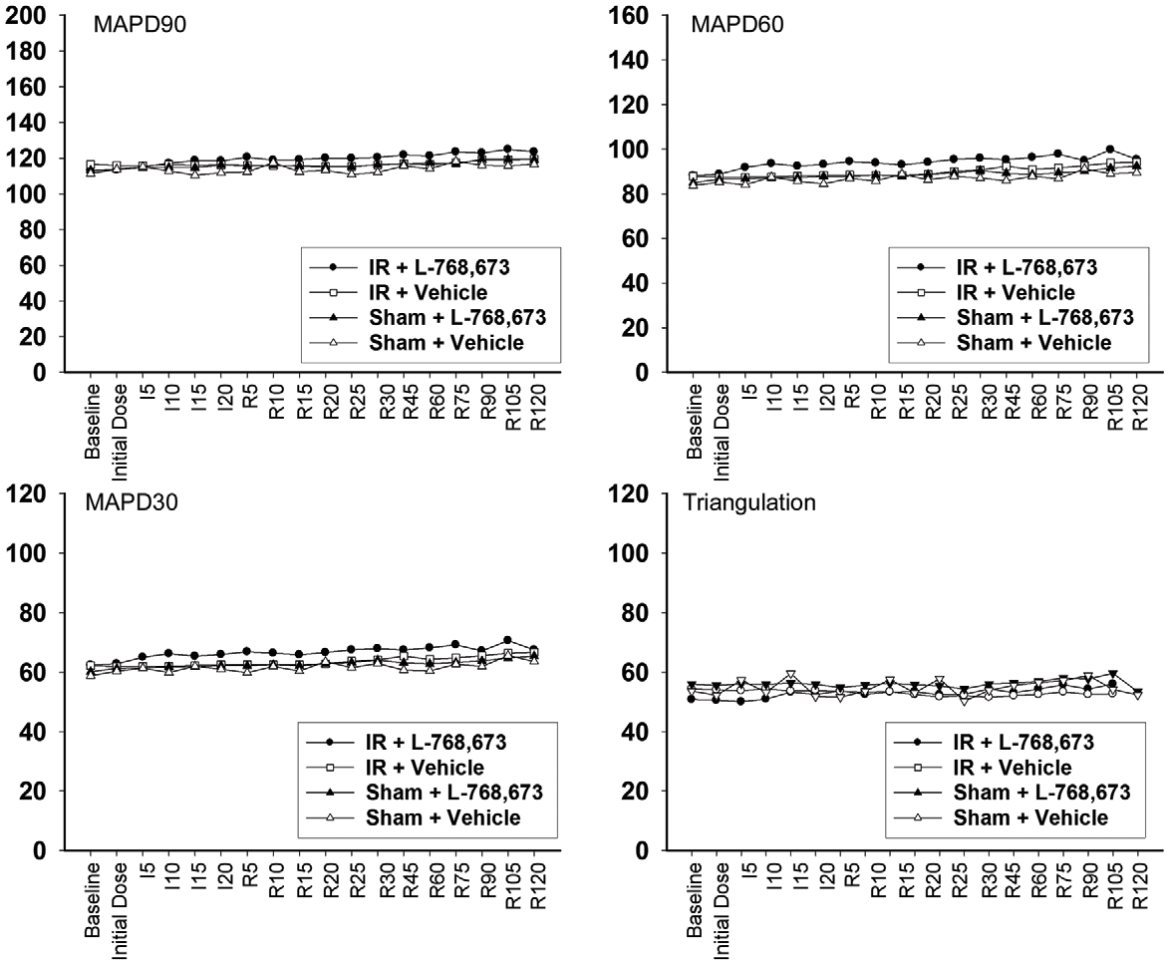
Epicardial MAPD90/60/30 and triangulation (MAPD90 - MAPD30) recorded from the remote zone (basal). Results were means ± STD. There were no significant differences between groups.

In the interval between reperfusion for 25 min (R25) and R90, the MAPD90 and MAPD60, but not the MAPD30, of the ischemia/reperfusion zone were significantly prolonged in the IR+L-768,673 group, when compared with the IR+vehicle group (Figure 2A). This denoted that in the presence of L-768,673, the reperfusion-induced prolongation of both the MAPD90 and MAPD60 was more evident and the return of the MAPD90 and MAPD60 to baseline was delayed; however, the MAPD30 did not show the same trend. This deviation led to an increase of triangulation (MAPD90–MAPD30) in the interval between R45 and R75, compared with the IR+vehicle group (Figure 2A). The MAP waveform of the IR+L-768,673 group was characteristic of a slowing of the fast repolarization (Figure 2B). Whereas, remote zone MAPDs recorded in all of the four groups exhibited mild, insignificant fluctuations (Figure 3).

The main effects of L-768,673 on MAPD90, MAPD30 and triangulation were further examined (Table 1). It was found that with the exclusion of the influence of ischemia/reperfusion, L-768,673 exerted independent impact on MAPD90 only at the time points R30, R45, R60, and R75, on triangulation only for the time points R45, R60, and R75; whereas, it exerted no independent impact on MAPD30. This finding perfectly demonstrated that I_Ks_ currents activated slowly and peaked during the late phase 3 and that blockade of I_Ks_ influenced only MAPD90, a surrogate of MAPD, with no effect on MAPD30, which represented the early phase of repolarization (phase 2 to the beginning of phase 3). The reperfusion after transient ischemia and I_Ks_ blockade by L-768,673 had a synergistic effect on prolongation of the MAPD90 and on the increase of triangulation in the interval between R45 and R75.

**Table 1:** The P value of main effects and interaction effects of I/R or sham operation and L-768,673 or vehicle on MAPD and triangulation.

	MAPD90			MAPD30			Triangulation		
Timepoint	I/R	L-768,673	Interaction	I/R	L-768,673	Interaction	I/R	L-768,673	Interaction
Baseline	0.714	0.353	0.929	0.210	0.900	0.980	0.094	0.178	0.887
Initial dose	0.272	0.109	0.838	0.101	0.123	0.432	0.262	0.301	0.402
I5	0.000*	0.572	0.857	0.700	0.843	0.500	0.005*	0.622	0.534
I10	0.055	0.861	0.826	0.001*	0.946	0.960	0.126	0.852	0.794
I15	0.000*	0.477	0.355	0.000*	0.917	0.869	0.000*	0.414	0.147
I20	0.000*	0.435	0.206	0.000*	0.720	0.858	0.000*	0.299	0.051
R5	0.000*	0.848	0.126	0.097	0.313	0.158	0.000*	0.348	0.980
R10	0.000*	0.187	0.901	0.000*	0.590	0.372	0.205	0.279	0.665
R15	0.000*	0.633	0.870	0.000*	0.449	0.449	0.540	0.848	0.575
R20	0.000*	0.058	0.368	0.000*	0.431	0.747	0.021*	0.177	0.561
R25	0.000*	0.111	0.053	0.000*	0.241	0.757	0.004*	0.344	0.088
R30	0.000*	0.021*	0.164	0.000*	0.262	0.100	0.001*	0.078	0.528
R45	0.000*	0.002*	0.021*	0.000*	0.065	0.478	0.038*	0.011*	0.044*
R60	0.001*	0.001*	0.009*	0.000*	0.096	0.564	0.246	0.003*	0.009*
R75	0.012*	0.007*	0.052	0.008*	0.248	0.831	0.422	0.010*	0.021*
R90	0.411	0.087	0.411	0.004*	0.150	0.689	0.145	0.195	0.523
R105	0.343	0.663	0.242	0.000*	0.257	0.988	0.000*	0.798	0.431
R120	0.349	0.108	0.916	0.000*	0.661	0.291	0.000*	0.026	0.993

I/R = Ischemia/reperfusion, interaction = interaction effect of ischemia/reperfusion and L-768,673, Triangulation = MAPD90–MAPD30.

P<0.05 highlighted by *.

### Influence of ischemia/reperfusion and L-768,673 on ventricular arrhythmias

A comparison of the occurrence of ventricular tachycardia, ventricular fibrillation, and total premature ventricular beats (PVB) did not show any significant differences between the groups (Table 2). The whole reperfusion period was then stratified by the presence or absence of a significant difference in triangulation between the IR+L-768,673 and the IR+vehicle groups into three consecutive intervals. Intervals 1, 2 and 3 corresponded to the interval from the initiation of reperfusion to R30, the interval from R30 to R75, and the interval from R75 to R120 respectively. PVBs in the IR+L-768,673 group were found to be significantly increased (4.6-fold) in Interval 2, compared with those in the IR+vehicle group, as shown in Table 2. This finding was in agreement with the significant triangulation synergistically induced by reperfusion and I_Ks_ blockade.

**Table 2:** Ventricular tachycardia (VT), ventricular fibrillation (VF), and total premature ventricular beats (PVB) of the ischemia/reperfusion (IR)+L-768,673 and IR+vehicle groups.

Group	VT*	VF* ^, ^ ‡	Total PVB	PVB in Interval 1§	PVB in Interval 2§	PVB in Interval 3§
IR+L-768,673	4/8	9/15	67 (14, 714.5)	10.5 (5.75, 30.0)	14.0 (9.5, 74.0)^**^	2.0 (0.0, 20.0)
IR+vehicle	1/8	9/15	60.5 (30.5, 131.5)	57.5 (16.8, 520.3)	2.5 (0.5, 10.0)	0 .0 (0.0, 2.0)

*All of the VTs and VFs occurred within 30 min of reperfusion.

‡ Animals that developed sustained VF were included in the analysis of VF, but were excluded from the analysis of other electrophysiological data.

§ Intervals 1, 2 and 3 were the interval from the initiation of reperfusion to R30, the interval from R30 to R75, and the interval from R75 to R120 respectively.

PVB data is expressed as median (25^th^ percentile, 75^th^ percentile). ^**^P<0.05 vs. IR+vehicle. The median was increased by 4.6-fold in Group IR+L-768,673. No significant differences between VT, VF, total PVB, PVB in Interval 1 and PVB in Interval 3 were detected between the groups.

### Effect of transient and chronic ischemia on MAPD shortening induced by epinephrine

**Figure 4:**
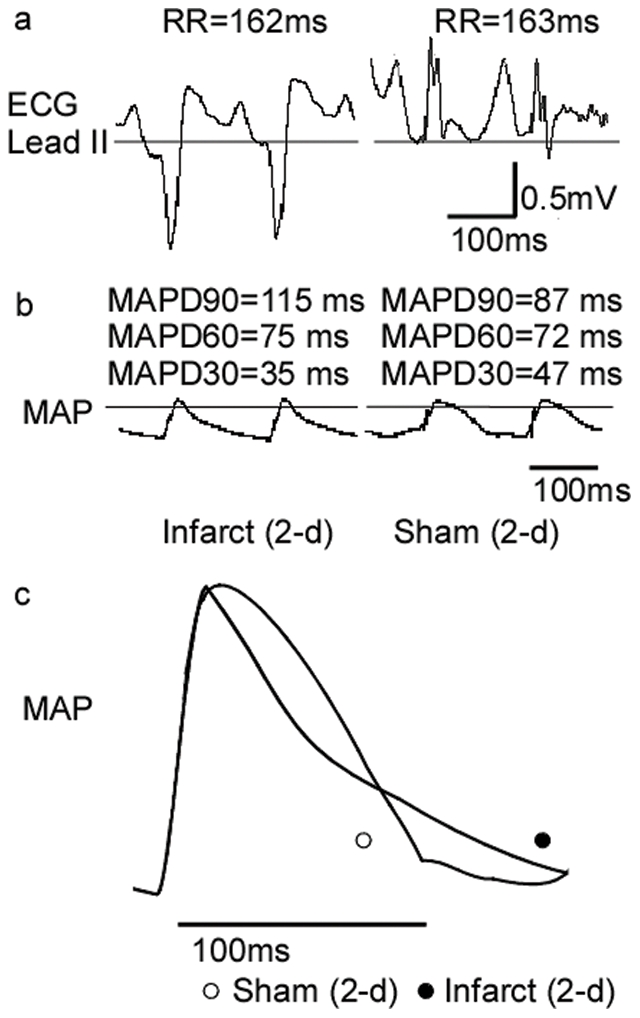
Comparison of waveforms between the Sham (2-d) and Infarct (2-d) groups after bolus injection of epinephrine. The only morphological differences in peri-infarct MAP were identified in the Infarct (2-d) group. (**A**) ECG Lead II waveforms showing that both groups had a comparable degree of heart rate increase. (**B**) MAP recorded at the peri-infarct epicardial zone showing that after the adrenergic challenge, the MAP of the Infarct (2-d) group had a dramatic shortening of MAPD30, whereas the MAPD90 was only minimally changed. (**C**) Direct overlapping of MAP showing that the MAP of the Infarct (2-d) group demonstrates more prominent triangulation.

The adrenergic stimulation triggered by bolus injection of epinephrine consisted of an increase heart rate (HR), acceleration and shortening of the MAPD90, MAPD60, and MAPD30 (Table 3). Concomitantly, the increase in MAP triangulation made the ventricles more vulnerable to ventricular arrhythmias, which partially explained why adrenergic stimulation induced ventricular arrhythmias in a normal heart. Moreover, the shortening of the peri-infarct epicardial MAP30 in the Infarct (2-d) group was as prominent as in other groups, whereas the peri-infarct epicardial MAPD90 of this group was only minimally changed. As a result, triangulation of the peri-infarct epicardium increased to the greatest extent in the Infarct (2-d) group (Figure 4).

**Table 3:** Comparison of MAP data recorded at the peri-infarct zone and heart rate (HR) in animals subjected to ischemia and healing, infarction, or sham operation.

Peri-infarct zone	Healing (2-d) (n = 6)	Infarct (2-d) (n = 8)	Sham (2-d) (n = 7)	Healing (5-d) (n = 7)	Infarct (5-d) (n = 7)	Sham (5-d) (n = 8)
**First operation**						
HR _pre-op_ (bpm)	294±8	306±13	292±6	293±7	297±9	287±13
MAPD30_ pre-op_ (ms)	70.2±4.0	70.8±3.7	70.8±4.1	70.1±2.8	69.9±3.1	70.5±3.0
MAPD60 _pre-op_ (ms)	98.8±3.5	101.1±3.5	100.6±4.4	99.7±3.9	99.3±4.1	100.7±3.5
MAPD90 _pre-op_ (ms)	120.7±4.1	122.0±4.3	123.0±5.1	121.7±4.6	121.3±5.3	123.3±4.8
Triangulation _pre-op_ (ms)	50.5±2.2	51.2±3.9	52.2±4.0	51.6±4.5	51.4±4.7	52.7±4.3
**Second operation**						
HR _post-op_ (bpm)	300±11	292±4	309±13	298±7	294±10	295±10
MAPD30 _post-op_ (ms)	66.1±6.9	66.6±4.0	67.8±3.4	68.9±3.4	70.6±5.0	69.6±3.8
MAPD60 _post-op_ (ms)	92.9±8.2	95.2±3.7	96.5±5.6	97.8±3.0	100.3±3.5	99.3±3.5
MAPD90 _post-op_ (ms)	118.7±4.3	123.6±3.2	118.7±4.1	120.4±2.1	125.9±3.6	120.6±2.3
Triangulation _post-op_ (ms)	52.6±4.5	57.0±6.1	51.9±3.9	51.6±4.3	55.2±8.5	51.1±4.2
**Epinephrine injection**						
HR _epi i.v._ (bpm)	382±14*	382±11*	378±11*	363±8*	366±6*	370±9*
HR acceleration (%)	27.3	30.8	22.3	21.8	24.5	25.4
MAPD30 _epi i.v._ (ms)	52.3±3.9*	36.2±6.6*	45.4±5.9*	54.1±3.8*	52.5±5.4*	50.4±4.9*
MAPD60 _epi i.v._ (ms)	73.8±6.9*	73.2±13.4*	64.3±6.2*	77.1±7.3*	74.4±6.1*	71.9±5.8*
MAPD90 _epi i.v._ (ms)	90.7±4.8*	114.3±9.4* #	90.9±7.4*	92.3±6.4*	94.0±2.6*	90.5±5.8*
Triangulation _epi i.v._ (ms)	38.3±4.0*	78.1±11.3* #	45.5±6.9*	38.2±5.3*	41.5±7.6*	40.1±5.1*
MAPD90 shortening (ms)	28.0±7.7	10.6±7.2#	28.9±8.3	28.1±5.0	31.9±2.2	30.1±5.6

Pre-op corresponds to data recorded at the first operation; post-op to data at the second operation; epi i.v. to data at the peak stimulation of epinephrine. Triangulation = MAPD90–MAPD30; MAPD90 shortening = MAPD90_post-op_−MAPD90 _epi i.v._; HR, heart rate; adr, epinephrine; bpm, beats per minute; i.v., intravenous injection.

*P<0.05 vs. post-op;

# P<0.05 vs. infarct (2-d).

### Influence of transient and chronic ischemia on adrenergic stimulation-induced ventricular arrhythmias

**Figure 5:**
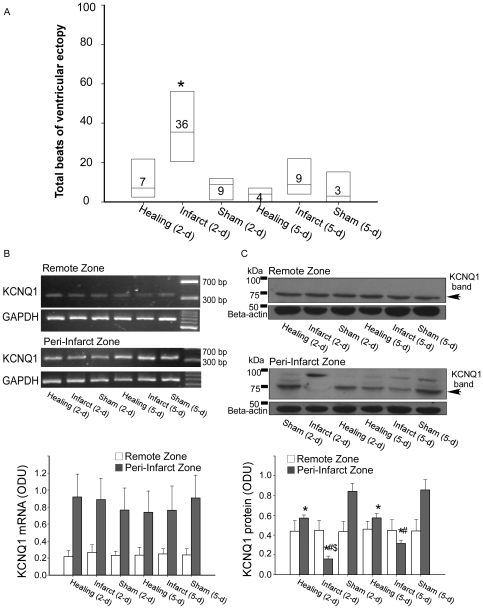
Comparison of ventricular premature beats and KCNQ1 expression between groups. (**A**) Comparison of the total premature ventricular beats (PVBs) between the groups within 10 min after bolus injection of epinephrine. Results are presented in a box plot format (n = 6–8) where boxes indicate the 25–75% interval along with the median of the data. * P<0.05 vs. the other groups. (**B**) Reverse transcription-polymerase chain reaction (RT-PCR) of KCNQ1 mRNA levels. Top: Examples of KCNQ1 mRNA with samples harvested from the peri-infarct zone and remote zone of the Healing (2-d; n = 6), Infarct (2-d; n = 8), Sham (2-d; n = 7), Healing (5-d; n = 7), Infarct (5-d; n = 7), and Sham (5-d; n = 8) groups of rabbit hearts. Bottom: mean KCNQ1 mRNA band intensities. (**C**) Western blot analysis of membrane-associated KCNQ1 protein levels. Top: Representative immunoblot results showing membrane KCNQ1 protein (∼75 kDa) with samples harvested from the peri-infarct zone and remote zone of the six groups of rabbit hearts. Bottom: mean membrane KCNQ1 protein band intensities. * P<0.05 vs. Sham (2-d) and Sham (5-d) respectively; # P<0.05 vs. Healing (2-d) and Healing (5-d) respectively; $ P<0.05 vs. Infarct (5-d).

Ventricular fibrillation was not induced in any of the animals subjected to transient or chronic ischemia, or in any of the corresponding control animals, when they were challenged with adrenalin. There were five episodes of ventricular tachycardia, which lasted for a total duration of 49 s, all of which occurred in 2/8 animals in the Infarct (2-d) group, although nonparametric statistical analysis showed no significance (Table 4). However, the Infarct (2-d) group was shown to be more vulnerable to ventricular arrhythmias than any of the other groups after pooling together total ventricular ectopy, with the exception of runs of ventricular tachycardia (Figure 5A).

**Table 4:** Comparison of episodes of ventricular tachycardia within 10 min of bolus injection of epinephrine.

Ventricular Tachycardia	Healing (2-d)	Infarct (2-d)	Sham (2-d)	Healing (5-d)	Infarct (5-d)	Sham (5-d)
Incidence	0/6	2/8	0/7	0/7	0/7	0/8
Episodes	0	5	0	0	0	0
Sum of duration(s)	0	49	0	0	0	0

No significant differences were identified between the groups; however, the runs of ventricular tachycardia occurred exclusively in the 2-d infarct group.

### Effect of transient and chronic ischemia on expression of I_Ks_ subunits

KCNQ1 mRNA expression remained unchanged in both the peri-infarct zone and the remote zone in our RT-PCR experiments (Figure 5B). However, KCNQ1 protein expression showed a different pattern in the peri-infarct zone under different ischemic protocols. KCNQ1 protein expression was greatly downregulated in the peri-infarct zone following ischemia for 2 d, and its expression in the peri-infarct zone increased again by approximately 2-fold by day 5. Transient ischemia was associated with a milder, but significant, impact on KCNQ1 protein expression, although no differences were identified between the Healing (2-d) and Healing (5-d) groups (Figure 5C). The steep downregulation of KCNQ1 protein expression (approximately 80%) might have severely compromised the repolarization reserve of the peri-infarct zone cardiomyocytes, which would in turn have led to an increased incidence of ventricular arrhythmias and greater triangulation of MAP in the Infarct (2-d) group under adrenergic stimulation. If the loss of the KCNQ1 protein subunit failed to exceed a certain threshold (for example, approximately one-third of the normal range shown in this study), the repolarization reserve was still able to function well and could at least compensate for a single factor that compromised other outward currents (that is, in this study, adrenergic stimulation inhibiting I_Kr_). Not surprisingly, the effect on KCNQ1 protein expression following transient or chronic ischemia in the remote zone was negligible, which was consistent with the unaffected MAPs in this zone.

KCNE1 mRNA levels were analyzed by RT-PCR; no significant differences were identified between groups (data not shown). Unfortunately, KCNE1 protein bands were very weak, which was consistent with the known low level expression of this protein in the rabbit [10]; therefore, conclusions regarding the protein expression levels of this subunit were not definitive.

## Discussion

### Repolarization reserve and ischemia/reperfusion-related ventricular arrhythmias

Even though it is widely acknowledged that the complications of cardiac ischemia, such as ATP deficiency, elevation of [K^+^]o, local acidosis, and lysophosphatidylcholine accumulation [11], amongst others lead to ischemia-induced arrhythmias and cardiac injury, the effect of ischemia and subsequent reperfusion on cardiac repolarization ion current has not been extensively explored. It has been shown that activation of I_KATP_ by low levels of ATP played an important role in the decrease in APD during ischemia [12]. On the other hand, the outward currents responsible for repolarization under normal conditions (for example I_K1_) were inhibited by low intracellular ATP levels [13]. With the restoration of blood flow, ischemic components were gradually cleared from the local milieu, which also brought about a subsequent transmembrane ion current variation that remains to be further elucidated.

The prolongation of MAPD in the early phase of reperfusion, which was reported previously by Bes et al. and Ducroq et al. *in vitro* [6], [7], was confirmed in our study *in vivo*. The careful use of controls in this study also demonstrated that blockade of I_Ks_ by L-768,673 prolonged both the MAPD90 and MAPD60 in the interval between R30and R90, which provided evidence of the increased contribution of the I_Ks_ current in repolarization currents. As previously demonstrated [2], blockade of I_Ks_ failed to lengthen normal ventricular muscle APD. In contrast, under conditions of ischemia and reperfusion, the independent effect of L-768,673 on MAPD90 prolongation at R30, R45, R60, and R75 revealed that the contribution of I_Ks_ to repolarization was markedly increased in this period. A similar increase in the contribution of I_Ks_ during this period was also shown by the synergistic effect of both ischemia/reperfusion and L-768,673 on MAPD90 prolongation at R45 and R60. Therefore, it may be inferred that I_Ks_ serves as an important repolarization reserve current and becomes the main repolarization current rather than I_Kr_ in early reperfusion. The administration of L-768,673 in this situation led to an increase in ventricular arrhythmias, which was a consequence of impairment of the repolarization reserve.

### Triangulation and predisposition to ventricular arrhythmias

The association between proarrhythmic effects and action potential prolongation may not be causal. MAPD90 is primarily the sum of the plateau and the fast repolarization phase of the action potential; therefore, MAPD90 can be lengthened by prolonging the plateau or by prolonging the fast repolarization phase, referred to as delaying or slowing of the fast repolarization respectively. The latter, described as triangulation of the action potential, is a reliable biomarker for proarrhythmia that was first described in 2001 by Hondeghem et al. in an automated Langendorff-perfused isolated rabbit heart preparation [14]. It is important to stress that lengthening of the MAPD90 without triangulation is not proarrhythmic, but rather antiarrhythmic [15].

Triangulation resulted from a reduction in outward repolarizing currents and/or an increase in depolarizing inward currents during fast repolarization; in MAP recordings, dispersion of repolarization may also contribute to triangulation. These conclusions have been previously confirmed with numerous experimental protocols performed on isolated tissues, perfused hearts, and experimental animals by other researchers [15], [16], [17], [18], [19], [20].

Triangulation was found to be increased in the interval between R45 and R75 in the IR+L-768,673 group; within this period, there was also a statistically significant synergistic effect of ischemia/reperfusion and L-768,673 on the increase in triangulation. Both these observations were consistent with the increased incidence of ventricular arrhythmias. In the transient and chronic ischemia protocol, increased triangulation of the peri-infarct epicardium under adrenergic stimulation in the Infarct (2-d) group was also observed, which increased the propensity for ventricular arrhythmias.

It has recently been reported that sarcoplasmic reticulum calcium (Ca^2+^) handling alteration resulted in a decrease in phase 2 of the action potential and was the key determinant of action potential triangulation in mice [21]. This may be largely due to the absence of I_Ks_ in species such as the rat and mouse [22], in which the APD is much shorter than in bigger animals such as the guinea pig, rabbit, dog, and humans. Transient outward potassium current (I_to_) and Ca^2+^ handling were found to be the main current that determined action potential in these species. However, the importance of sarcoplasmic reticulum Ca^2+^ handling in action potential triangulation of large animals still needs to be carefully evaluated.

### KCNQ1 expression and cardiac ischemia

There have been a limited number of reports regarding the effect of ischemia on I_Ks_ amplitude and KCNQ1 expression. Selected examples of other animal models of heart disease, for which remodeling of I_Ks_ has been described and for which information on the underlying changes in channel subunit expression was available, are listed in Table 5. This comparison illustrates that an apparently similar phenotypic change can have divergent molecular mechanisms. To date, neither KCNQ1 nor KCNE1 protein expression levels under conditions of ischemia have been reported.

**Table 5:** Divergent molecular mechanisms for potassium channel remodeling in animal models of heart disease.

Species/Region	Etiology	Functional changes	Molecular changes at the protein level		Molecular changes at the mRNA level		Reference No.
			KCNQ1	KCNE1	KCNQ1	KCNE1	
Rabbit/ventricle	AV block with ventricular pacing						
	Tachycardia	↓	↓	↓*	↓	↓	[24]
	Bradycardia	↓	↓	↓*	↓	↓	[24]
Rabbit/ventricle	Pacing-induced heart failure	↓	-	ND	-	-	[39]
Dog/ventricle	AV block-induced hypertrophy	↓	↓	↓	↓	↓	[40]
Dog/ventricle	Chonic ICM by microembolizations	↓	-‡	↓	ND	ND	[41]
Dog/ventricle	Myocardial infarction	↓	ND	ND	↓	↓	[8]

AV, atrioventricular; ICM, ischemic cardiomyopathy; ND, not determined; -, no change.

*Weak bands limited the reliability of the measurement.

‡ KCNQ1.2, a truncated isoform of canine KCNQ1, was increased and may suppress I_Ks_ in a dominant-negative fashion.

In this study, membrane KCNQ1 protein expression was severely depressed by chronic ischemia and moderately downregulated by transient ischemia (20 min ischemia in our protocol). However, KCNQ1 protein expression did recover to some extent by d5 after chronic ischemia, and no significant difference was observed between cardiac KCNQ1 protein expression at d2 and d5 after transient ischemia. As a pore-forming subunit of the I_Ks_ channel, decreased KCNQ1 protein levels could lead to a rapid decrease in I_Ks_ current, which has been well documented in human Romano-Ward Syndrome (LQT1 subtype) gene research [23] and in ion channel subunit remodeling research in animals exposed to long term arrhythmias [24]. Consistent with previous studies, our results confirmed the strong relationship between low KCNQ1 protein levels and diminished repolarization reserve, which resulted in triangulation of the action potential and an increased incidence of ventricular arrhythmias after adrenergic stimulation. The fact that only a dramatic decrease in KCNQ1 protein levels was associated with increased arrhythmic incidence provided evidence that downregulation of the I_Ks_ channel subunit may be complicated by multiple compensative mechanisms in the cardiomyocyte, such as potent repolarization reserve comprised of various potassium channels, diminished Ca^2+^ current minimizing Ca^2+^ overload, which were not addressed in our experiments [25].

No significant changes in KCNQ1 or KCNE1 mRNA expression levels were identified. The reasons for the discrepancy between observed changes in KCNQ1 protein levels and unchanged mRNA expression levels in this study are unclear, but several explanations can be offered. Firstly, changes in protein expression levels may fall behind changes in mRNA expression levels for a period of time that ranges from minutes to days. In this and previous studies, limited time points were chosen, which may prevent the detection and interpretation of dynamic changes in chemical biomarker levels. Secondly, changes in mRNA expression levels may not reflect changes in the functional protein subunit levels, because infarction may affect many processes, which include protein translation, subunit modification, assembly, processing, and trafficking. Known KCNQ1 subunit modifications to date include classical phosphorylation and dephosphorylation [26], [27], glycosylation and deglycosylation [28], [29], and most recently, ubiquitylation [30]. This discrepancy provides a clue that post-translational mechanisms may play a more important role in I_Ks_ subunit suppression.

### Limitation of study

In addition to APD and triangulation, other variables had been identified in the MAP waveform that predict the pro-arrhythmic effect of drugs or pathophysiological conditions in a comprehensive manner under different experimental protocols. Among them, triangulation, reverse use dependence, instability and dispersion of ventricular repolarization, together with the cardiac wavelength are powerful proarrhythmic predictors [31]. APD and triangulation were applied in this study to identify the association between ventricular arrhythmias and repolarization reserve compromise; other as yet unidentified predictors may also play an important role. Subunit interactions, whose importance in ion channel function regulation is well-established [32], were not addressed in the current study due to low KCNE1 protein expression level. The roles of other ion channels in cardiac ischemia-reperfusion induced ventricular arrhythmias were not exhaustively covered in this study, even if control groups had been well set up in the protocols.

### Conclusions

The results of this study highlight the importance of maintaining an intact repolarization reserve for the prevention of ventricular arrhythmias both in cardiac ischemia/reperfusion and in the infarcted heart under adrenergic challenge. I_Ks_ served as a protective current in securing action potential repolarization, and the reduction of I_Ks_ that resulted from downregulation of the I_Ks_ channel subunit protein contributed to ventricular arrhythmias.

## Materials and Methods

### Animal preparation

All animal experiments were approved by the Animal Care Committee of Sun Yat-sen University, Guangzhou, China, and all investigations conformed to the Guide for the care and Use of Laboratory Animals published by the United States National Institutes of Health. New Zealand white rabbits (1.5–2.0 kg) of either sex were purchased from the Provincial Medical Laboratory Animal Center (Guangzhou, China).

### In vivo model of rabbit coronary artery occlusion/reperfusion

In a modification of a previously published procedure [33], Rabbits were anesthetized by subcutaneous administration of ketamine (40 mg/kg body weight) and Xylazine (8 mg/kg body weight). An endotracheal tube was inserted and used for ventilation with room air at 38 strokes per min and a stroke volume of 7 ml/kg with a positive end-expiratory pressure of 2 KPa applied. Body temperature was maintained by an appropriate heating lamp. Arterial blood pressure was recorded by left jugular artery intubation. The heart was exposed by performing a midline thoracotomy. A ligature was placed around the left circumflex coronary artery at a distance of 10 mm from its origin at the coronary groove. The ends were exteriorized and passed through a tapered polyethylene tube. After the animal had been allowed to stabilize for 15 min, coronary artery occlusion was achieved by pressing the tube against the heart muscle while pulling on the ligature, followed by clamping the tube with a hemostat; this was accompanied by immediate pallor of the left ventricular free wall, a marked drop in blood pressure, and immediate ST segment elevation in the ECG waveform. Reflow was initiated by releasing the ligature, which was accompanied by immediate hyperemia of the left ventricular free wall as well as a marked increase of blood pressure.

### Electrophysiological parameters

ECG Lead II was recorded continuously with subcutaneous needle electrodes. A spring-loaded epicardial Ag-AgCl electrode was made as described previously [34]. Epicardial monophasic action potentials (MAPs) were recorded at various locations in the ventricular epicardium using the electrode at specific time points intermittently throughout the experiment. All data were recorded using a TME BL420 multichannel recorder (TME Technology, Chengdu, China). The diagnoses of ventricular arrhythmias were made in accordance with the Lambeth convention [35]. The MAP parameters measured included: monophasic action potential duration (MAPD) at 30%, 60%, and 90% of repolarization (MAPD30, MAPD60, and MAPD90, respectively). Each parameter was average of at least 5 measurements. The slowing of repolarization in MAP was assessed quantitatively by triangulation, which was calculated as the difference between MAPD90 and MAPD30 in milliseconds (MAPD90 - MAPD30).

### Drugs

An appropriate portion of L-768,673, a highly selective I_Ks_ blocker that has been extensively studied in *in vitro* and *in vivo* models [36], [37], [38], was first dissolved in 100% ethanol at a concentration of 1 mg/ml, followed by suspension in 0.9% saline to yield a final concentration of 5 mg/l. Then the formulation was dosed via the left marginal vein with an initial dose of 1 µg/kg for 30 min (to ensure complete drug equilibration) while a maintaining dose of 0.5 µg/kg for 2 h [37], [38]. Control group animals were dosed with an equal volume of vehicle solution, i.e. 100% ethanol dissolved in 0.9% saline at a concentration of 0.5% (v/v).

### Reverse transcription-polymerase chain reaction

Total RNA was isolated from samples with TRIzol reagent (Invitrogen Corporation, Carlsbad, CA, USA), followed by chloroform extraction and isopropanol precipitation. cDNA was synthesized by reverse transcription. PCR was performed using the cDNA templates in a reaction buffer containing a corresponding primer (Table 6) and Taq DNA polymerase (Invitrogen Corporation). The PCR products were then separated using agarose gel electrophoresis. The bands were visualized under ultraviolet light following ethidium bromide staining. PCR product integrity was quantitated using the UVItec gel system (UVItec Limited, Cambridge, United Kingdom) and were normalized using the corresponding GAPDH data.

**Table 6:** Details of PCR.

Gene		Primer sequences (5′–3′)	PCR product length (bp)	Annealing temperature (°C)	Cycles
KCNQ1	Forward	GCCGCAGCAAGTATGTCG	317	58.6	33
	Reverse	CCTTCTCAGCAGGTACACGA			
KCNE1	Forward	CCGTGATGCCCTTTCTGACC	263	62	34
	Reverse	GTACGCCCTGTCTTTCTCCTG			
GAPDH	Forward	GATCCATTCATTGACCTCCACTA	683	58.6	30
	Reverse	CACCACCTTCTTGATGTCGTC			

### Immunoblot analysis

Membrane fractions were prepared using the BioVision Plasma Membrane Protein Extraction Kit. Protein samples were separated with 8% SDS-PAGE using a minigel system (Bio-Rad Laboratories, Hercules, CA, USA). Proteins were transferred onto an Immobilon-P polyvinylidene fluoride membrane (0.45 µm pore size; Millipore Corporate, Billerica, MA, USA) in 25 mM Tris base, 200 mM glycine, and 20% methanol using the Mini Trans-Blot transfer apparatus (Bio-Rad Laboratories, Hercules, CA, USA). Following the transfer, the membranes were incubated for 2 h at 4°C in blocking buffer (PBS containing 5% nonfat milk powder and 0.1% Tween 20). The membrane was incubated for 18 h at 4°C in blocking buffer containing primary antibody. Antibodies against KCNQ1 (sc-10646; goat) and KCNE1 (sc-16796; goat) were purchased from Santa Cruz Biotechnology, Santa Cruz, CA, USA. After membranes were washed, the bound antibody was detected using horseradish peroxidase-conjugated donkey anti-goat IgG secondary antibody (Santa Cruz Biotechnology, Santa Cruz, CA, USA) in blocking buffer for 1 h, followed by detection with the BeyoECL Plus detection system (Catalog # P0018). The immunoblots were exposed on Fuji film. Band signals were detected and quantified with laser scanning and Image J software. The immunoblot band intensity values reported herein correspond to background-subtracted optical density units (ODUs) normalized to β actin signal intensity for the same sample.

### Study protocol 1

**Figure 6:**
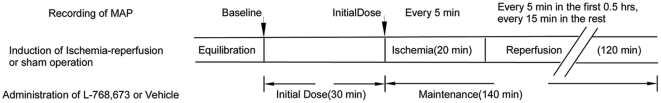
Study protocol 1 of experiments. Monophasic action potentials were recorded after the equilibration at both the middle of the infarct zone and the unaffected zone of the epicardium. For L-768,673, The infusion rate of the initial dose was 0.5 µg/kg/h for 30 min, and the maintenance rate was 0.25 µg/kg/h for two hours. MAP duration data were expressed as MAPD90/60/30_Baseline, Initial Dose, I5, I10, I15, I20, R5, R10, R15, R20, R25, R30, R45, R60, R75, R90, R105, R120_, which stood for MAPD90/60/30 recorded at baseline, after initial dose, at ischemia for 5 min, 10 min, 15 min, 20 min and at reperfusion for 5 min, 10 min, 15 min, 20 min, 25 min, 30 min, 45 min, 60 min, 75 min, 90 min, 105 min, 120 min, respectively.

In protocol 1, which was based on a 2×2 factorial design, 42 animals were randomized to receive either 20 min of left circumflex coronary artery occlusion followed by 120 min of reperfusion (IR) or a sham operation for the same duration (Sham), with either intravenous administration of L-768,673 or a similar volume of the vehicle. The initial sample sizes of the four groups were as follows: IR+L-768,673, n = 15; IR+vehicle, n = 15; Sham+L-768,673, n = 6; Sham+vehicle, n = 6 (Figure 6). In accordance with the Lambeth conventions [35], seven animals in both the IR+vehicle group and the IR+L-768,673 group that developed sustained ventricular fibrillation were censored in the analysis of electrophysiological data because of the potentially unpredictable manifestations thereafter.

### Study protocol 2

In protocol 2, 56 animals were randomly divided into the following six groups: Healing (2-d) group (n = 10); Infarct (2-d) group (n = 10); Sham (2-d) group (n = 8); Healing (5-d) group (n = 10); Infarct (5-d) group (n = 10); and Sham (5-d) group (n = 8). The induction of ischemia and reperfusion were performed in a manner identical to the first protocol. Infarction was induced by direct tightening of the ligature. Several animals were excluded from the analysis because of the development of sustained ventricular fibrillation: in the Healing (2-d) group, 4/10; in the Infarct (2-d) group, 2/10; in the Healing (5-d) group, 3/10; and in the Infarct (5-d) group, 3/10.

**Figure 7:**
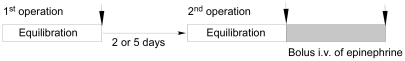
Study protocol 2 of experiments. Monophasic action potentials were recorded after the equilibration at both the peri-infarct zone and the unaffected zone of the epicardium during each open chest operation and at the time point of peak adrenergic excitation. MAP duration data were expressed as MAPD90/60/30_pre-op, post-op, epi i.v._, which stood for MAPD90/60/30 recorded at first operation, at second operation, and after the bolus intravenous injection of epinephrine, respectively.

The number of surgical survivors were 6/10 animals in the Healing (2-d) group; 8/10 from the Infarct (2-d) group; 7/8 from the Sham (2-d) group; 7/10 from the Healing (5-d) group; 7/10 from the Infarct (5-d) group; and 8/8 from the Sham (5-d) group. Following surgery, the surviving animals were housed in cages for 2 d or 5 d. At the end of the protocol, the chests of the anesthetized animals were reopened, and the animals received a 40 µg/kg intravenous bolus of 0.01% (w/v) epinephrine [16]. Monophasic action potentials (MAPs) in both the peri-infarct epicardial zone and the intact epicardial zone were recorded during each operation and after the administration of epinephrine stimulation (Figure 7). Cardiac tissues were harvested and distinguished as peri-infarct zone and intact zone or the counterpart in control groups.

### Data analysis and statistics

All quantitative data with normal distributions were expressed as means ± STD. Other data without normal distributions were expressed as medians with 25^th^ and 75^th^ percentile. Means were compared between groups with two-way ANOVA, followed by the SNK-t post hoc test. The main and interaction effects of operation (ischemia/reperfusion or sham operation) and drug treatment (L-768,673 or vehicle) were analyzed by two-way ANOVA using a General Linear Model.

The presence of any life-threatening ventricular arrhythmias (e.g., ventricular fibrillation, ventricular tachycardia) between groups were assessed with the Fisher's exact test. The number of premature ventricular beats between groups were expressed as median with 25^th^ and 75^th^ percentile and were compared by using the K independent nonparametric test, followed by two independent Kruskal-Wallis nonparametric tests.

Values of P<0.05 indicated statistical significance. All statistical tests were performed using SPSS statistics 17.0 (GraphPad Software Inc., San Diego, CA, USA).
